# Supplementary material for: Patterns of Intron Gain and Loss in Fungi
Source: PLoS Biol. 2004 Nov 30;2(12):e422. doi: 10.1371/journal.pbio.0020422 (PMC532390; doi:10.1371/journal.pbio.0020422)
Supplement: Table S1 — Also available at http://genes.mit.edu/NielsenEtAl/. (4.3 MB ZIP). [file pbio.0020422.st001.zip › NielsenEtAl/html/695.html]

AN4876.1.NCU05902.1.MG03313.1.FG06075.1


```
 CLUSTAL W (1.82) Multiple Sequence Alignments - Introns Inserted


Sequence 1: NCU05902.1	1359 aa
Sequence 2: FG06075.1	1348 aa
Sequence 3: MG03313.1	1357 aa
Sequence 4: AN4876.1	1280 aa
Alignment Length: 1438 aa
Number Identitical Residues: 459 aa
Alignment Score (without introns) 25727


MG03313.1 	MSDE----------------------------------------ETPNVNNGIQSIGDYT
NCU05902.1	MTQVPPETG--------------DSADTTPAPTIVTS-----PRARPKRSIGSSTIGTTT
FG06075.1 	MTDQSPEPSHEQARDLETQQPEHDAAPTSSEPVPPTNNDASDDGAHSSESESISKEGDVS
AN4876.1  	-------------------------------------------MDAPGATEASLSRVEPG
          	                                              .  . .  .     

MG03313.1 	ESSPQTPSQPPNVAVSDERAAESTLEKVASTPDEDGDESE---EEEDDEDDDDEDEEDDE
NCU05902.1	TSTATATG--TGTDGESGISVEEGLEVNGGRADGEGEETEDDDEDEDEDDDEDDDEEEEE
FG06075.1 	AKDVTPPGSNDGKASQDGDQDKEGGEEDDNDEDDDNDEEDDDDEDEDDEEDEDEEEEDEE
AN4876.1  	QGSPTRPSTHHRRP-HRQLHTETGDDDEEEQEDPNREREE---SMTETMSYYGEEEEDDE
          	      ..             :   :      * : :. :   .  :  .  .::**::*

MG03313.1 	EEEE----PRLKYTRLTQNLGAVYKNGDATSSFLVAGDKM0IIGTHSGNI0HAVQLPSFQ
NCU05902.1	EEED--EEPKLKYARLTEHLGPLYRNGDATSAFLTAGDKM0IVGTHNGNI~HVLQLPAFQ
FG06075.1 	DEEDDDEEPKLKYARLTQHLNGVYRNGDATSAFLVAGDKM0IVGTHNGNI0HIVQLPMFQ
AN4876.1  	EEEE----PHLKCSHLTKQLGSAYRNGDATSTFLAAGDKM0VLGTHNGNI0HVFSVPLFR
          	:**:    *:** ::**::*.  *:******:**.***** ::***.*** * ..:* *:

MG03313.1 	TLLVYHAHSASVTSVSISPCPPPVSSLRPDVLKAAPQNSE----TASLRRPASKHSEASP
NCU05902.1	SLRVYHAHSASVTSLSISPYPPPLPTFDPKPLTRVFSQTNSPLRPASASTESHHQTNPAS
FG06075.1 	SMRVYHAHSASVTSISISPYPPPLPTDIPETVSKHGAHSN-------ASISSSRQTDSSS
AN4876.1  	SLRVYHAHSATVTSISISPFPPPAPNLKHELATRFAENQS------------ASTKPFSS
          	:: *******:***:**** *** ..   .  .    : .               .  :.

MG03313.1 	AAASRKPREAPPVPNMPSNHIHIATSSMDGNVCVQSLMERRDVQLRNFARPVQAVALSPE
NCU05902.1	-AARKRDAQAHGVPNTPSNNIHIATSSMDGNVCVQSLIDVKDVTLRNFARPVQAVALSPD
FG06075.1 	PAPSRRPREVSQIPRTPSNDIYVATSSLDGNVCVQNLIDMKDVQLRNFARPVQTVALSPE
AN4876.1  	SGSIRGQSKPTSLPTTPSNSIYIASSSIDGNVCVASLLDTKDVLLRNFGRPVQAVALSPE
          	... :   :   :*  *** *::*:**:****** .*:: :** ****.****:*****:

MG03313.1 	YKSDKTYLSGGLAGSLILTVGGQTGKSN-STTIGTAAATASSWLGSIGLAGNAGKDTVLH
NCU05902.1	YKNDRTYLSGGLAGQLILTVGAPQGKST-ATTTG-AAAQAAGWLGNM-VGAGSGKDTVLH
FG06075.1 	FKTDRTYLSGGLAGQLILTAGGGPGRST-STTTGTAAATASGWLGSMGLGGNAGKDTILH
AN4876.1  	YKSDRTFISGGRAGELILTTGGKVGVSTNSTTLGGAAATASSWLGTFGLGGNTGKDTVLH
          	:*.*:*::*** **.****.*.  * *..:** * *** *:.***.:.:...:****:**

MG03313.1 	SGEGTISTIKWSLSGKYVAWLNEHGIKIMRSKIKLDSADADDAWKRIGHIDRPQSSEWDA
NCU05902.1	SGEGTINTIKWSGSGKYVVWLNEHGIKIMRTKLHLESADQEDAWKRIGHIDRPQTEEWDT
FG06075.1 	SGEGTISTIKWSLSGKYVVWLNEHGIKIMRSKLHLESADAEDAWKRVGHIDRPQTDEWET
AN4876.1  	SGEGAISTVKWSLSGKYVAWVNEEGIKIMRSNLHLDSVDSELAWKRVSHTDRPNRQGWEE
          	****:*.*:*** *****.*:**.******::::*:*.* : ****:.* ***: . *: 

MG03313.1 	MASAWRGRAEWMDEQAVEMDDNADTTLVGDSRSDQDSSGTLSPAAAKLKQQQSKHDKTIE
NCU05902.1	MASVWKGRAEWIDEQSIEPDE------PGKGQKEVPAS----PAAAILKQQHQKTEKKVE
FG06075.1 	MASVWKGRVEWIDEQAVEVDE------TDQSATEKSSS----PATEKLKGHALASKKGIE
AN4876.1  	MASVWKARAEWVDESLLESEH----------LTSNNTG-----GEASTPTQSTIIKERQE
          	***.*:.*.**:**. :* :.           ..  :.     .      :    .:  *

MG03313.1 	KLVVGWGGTIWVIHVHPGGMGVGKNAGEKSVGRAEIHQK2LRMDCIISGISLYTQSLLLI
NCU05902.1	RLLVGWGGTIWFIHVHPGGMGVGKHAGERSAGRAEIVKL2LRMDCIISGISLYTQNLLLV
FG06075.1 	RLLVGWGGTIWIIHVHPGGVGVGRHAGEKTIGRAEIVKI2LRMDCIISGISLYTQNLLLV
AN4876.1  	KLVVGWGGTVWVINVYPD--RPNKSNRDLRIGSVEVATI2LRTDCIISGISLYSPSHLVV
          	:*:******:*.*:*:*.    .:   :   * .*:    ** **********: . *::

MG03313.1 	LAYCLPEDEDEDDDEDGDGEKTPTQPRGHRSRPSAASSSSEPSGGIRRRQNNQPPELRLI
NCU05902.1	LAYCLPDENEDEEDD------NVIPS--HKHAASIASRASHPSGGIKRRQNNPPPELRLI
FG06075.1 	LAYCLPEDDDEDEGA------VSVSNSPDKRHKPNSSTGSQPSGGLPRRQNNQPPELRLI
AN4876.1  	LAYIEAENEPADEQIT------------------------------RRRPRGQEPELRII
          	***  .:::  ::                                  ** ..  ****:*

MG03313.1 	DLKSQSEIYKDGLSISRYERLSASDYHLGVLPAQNQEAVASS-KSALEALAGFGTDMWNA
NCU05902.1	DLTSQAEIDKDGLSVSRFERLTASDYHLGVLPARNVAASAAS-KGTLETLAGFGTDMLNV
FG06075.1 	DLNSQAEADKDSLSVSRYERLSSGDYHLGVLPARNAASAIASSRGALEAIAGIGTDMWNA
AN4876.1  	DIESKEEVSADTLAISRYENLTASDYHMSVLPPWKTNMPVSQ-RGALEALG---TGLWDA
          	*: *: *   * *::**:*.*::.***:.***. :     :. :.:**::.   *.: :.

MG03313.1 	-------AIKPKMLFSSGASIRSGHS-NDATSGSKVSLAGSSIRAPFKLQTQAVHPSLET
NCU05902.1	GLTGLNVALNPKSLFSSGASIKSRDS-DETPSN----YMGGINSALKGGHRTAIHQNLNK
FG06075.1 	-------AINPRSLFSSGASIRSRGS-GDDSSIR-GSTAGTIRPGTSRGLSPTVHSGLVK
AN4876.1  	-------TMYPARLFSSAASIRSSTSSGDRSSNRAPSTFASRRSVPEEPLAIEVQEVAES
          	       :: *  ****.***:*  *:.: .*    :  .             ::    .

MG03313.1 	PGPKIFMHSPYDCVLATKRDLGDHLTWLVEHQEYQAAWELLDEHPEIMATSPSVSDLVAT
NCU05902.1	PGIKIFIHSPYDCILATKRDLADHLGWLLERQQYRQAWELVDEHPEIVS-ATDRAS-ELG
FG06075.1 	PGVKIFIHSPYDCILATRRDLSDHLGWLLERQQYQRAWELLDEHPEIMAPAGERAN-DNT
AN4876.1  	SGPKIFVHSPYDCVAALKRDLADHLAWLIEHEKYAEAWKLLDEHPEAAGSSEGSDNVSIT
          	.* ***:******: * :***.*** **:*:::*  **:*:*****  ..:    .    

MG03313.1 	PTAERPTQVSDDLFDDTTSTVDAVSRAFNSSVAREKRRIGELWLQDLIEDNDWVKAGQIA
NCU05902.1	G-SETPDRAQNDDFDDETSSVIDGLRSHYSLAEKEKRRIGELWIHELVEANDWAQAGQIC
FG06075.1 	PPTPTLNQEASDEFNDDESVIDSQVRDFYSSAEKEKRRIGELWIQELIEENDWVSAGKIC
AN4876.1  	P--GRSQTSLGDLFVDDRSSITATDRGNVPAAVQEKRRIGEMWIEQLIRDNRWQEAARVC
          	          .* * *  * :    *   . . :*******:*:.:*:. * * .*.::.

MG03313.1 	AKVLGSSERWEKWIWTFAGADKFDEIVNYVPTEPMRPPIDGSVYEVMLGHYLREDKPRFR
NCU05902.1	GKVLGTPDRWEKWVWTFAGANKFDDIVNYIPTERTRPPIPGTIYEVVLGHYLQVSKPRFR
FG06075.1 	GQVLKTPDRWEKWVWTFAGAKRFDAITNYIPTKPMHPPLPSTIYEVVLGYYIQHDKPRFR
AN4876.1  	VQALSATSRWEHWAWTFIKENKFDEITSVIPVD-LRPSLSSEIYGAILEHYLTRDRQKFS
          	 :.* :..***:* ***   .:** *.. :*..  :*.: . :* .:* :*:  .: :* 

MG03313.1 	ELLDRWPVELFDANTIATTLENQLKYRDVREDSVEDGEKGRDWRIVMESLARLHEANGRL
NCU05902.1	ELLERWSSDLFDVATITTALENQLKYRDVREDSVEDGEVGRDWRIVMESLAKLHEASGRT
FG06075.1 	ELLDRWSPELFDVKTITTALENQLNYRDVREDSIDDGERGRDWKIVMESLARLHEAIGRY
AN4876.1  	ELFETWPFELFDIDSITTSIEEQLESEKMTPD-LEDG------RILIRCLARLYLTGGHY
          	**:: *. :***  :*:*::*:**: ..:  * ::**      :*::..**:*: : *: 

MG03313.1 	REALKCNIRLQDAENAFRLIREGHLADAVVDDIPSFILLRLPSGMETKMSLEEVEAATSE
NCU05902.1	KEALRCYIRLQDADSAMRLIKDAHLAEAVADDIPSFIGLRVPQDKLLKMSKEDLEQATSE
FG06075.1 	REALKCYIKLHDADSAFRLIRDNHLAEAVEDDIPSFIGLRVPPGKLDHMTAEELELATSE
AN4876.1  	TDALHCYIRIQDADAAMALVKDHHLLDSLSDDIPAFIMIRVSKEQLKSAPISELAEITAE
          	 :**:* *:::**: *: *::: ** ::: ****:** :*:.       . .::   *:*

MG03313.1 	AITLLVDEAQNGLVKPEVVVSQLQEREDLHAYLFFYIRGLWTGQGVAPHRHEGAA----E
NCU05902.1	AISLLVDEAQHGLVKPDLVVSQLQEK-DLKLYTFFYLRGLWRGEGIHEHTHE--------
FG06075.1 	AIILLVDEAQHGLLRPDVVVEQLLAQ-KLNLYIYFYFRGLWRGEGIQEHGGE--------
AN4876.1  	PIKLLASEAHTGIVPPDTVVRQLQTA-NKPLFLFFYLRALWRGESLSLEAEKPRRGRHRQ
          	.* **..**: *:: *: ** **    .   : :**:*.** *:.:  .  :   .   .

MG03313.1 	ARERMLDD-SRSLVDDFADLAVQMFATYDRSLLMEFLEHSLSYSFEK~AEKVCEEHNFIP
NCU05902.1	SLARLVLD-SRTMVDQFADLAVHLFAIYDQPLLNQFLRTSTAYAFEK0AAQECESRNYIP
FG06075.1 	NVDRLVMD-SQSLVDSFSDLAVHLFATFDRSLLMEYLKTSVSYTFEK0AVQECENFSYYD
AN4876.1  	AATKLAADEGKNLVDQFADTAAELFAQYERPLLMEFLQTSTSYSFDV~AVTICEQYRFTP
          	   ::  *..:.:**.*:* *..:** :::.** ::*. * :*:*:  *   **.  :  

MG03313.1 	ELVYLYAKTGQMKRALYLIIDRLADVSRAIAFAKTQDDPDLWEDLLNYSMDKPRFIRALL
NCU05902.1	ELVYLYSKTGQVKRALYLIIERLGDVSRAIAFAKEQDDPDLWEDLLEYSMDKPKFIRALL
FG06075.1 	ELVFLYSKTGQMKRALYLIIDRLKNVHKAIEFAKEQDDPDLWEDLLKYSMDKPSFIRGLL
AN4876.1  	ELIYLLSKMGQTKRALNLILSDLKDVSQAIAFAKSQDDPDLWEDLVDYSMDKPRFIHGLL
          	**::* :* ** **** **:. * :* :** *** **********:.****** **:.**

MG03313.1 	EEVGTAIDPITLVRRIPEGLEIPGLREGLTHIMKEHEIQHSISSGVARVLRSEVSGAQAT
NCU05902.1	EEVGTAINPITLVRRIPEGLQIPGLREGIRHIMKEHEIQYSISEGVSRVLRSEVAAAMNT
FG06075.1 	EQVGTAINPITVVKRIPEGLEIEGLREGLTHMMKEHEIQYSISSGVARVLRSEVATAQTE
AN4876.1  	VEAGTSIDPIKLVRRIPSGLEIEGLREGLTGLLREHDLQASISQGAAKVLQSEVAVGMNT
          	 :.**:*:**.:*:***.**:* *****:  :::**::* ***.*.::**:***: .   

MG03313.1 	LRSGQRRGVKFEV-APLGQTHMDANTTGRGGNDVADAAAAALDKALASAQGSAPVADPDA
NCU05902.1	LRNGQRKGVKFQVGVREGGEHVDVQPTDIPTQPLTEKPSSSGLASPAAPSQQPPPTPSTP
FG06075.1 	LRAGQRKGIKFEV-VQQDPEHVDIEVKDVPTD-------------PENPERNASHPSTKH
AN4876.1  	LRDGQRRGIKFNIIQESSKSDQVNDEAKAETD----------------SEKTPTPSRGSF
          	** ***:*:**::    .  .   :      :                ..  .. .    

MG03313.1 	SGAVAKIP-KSWTPGHCPKCLEAFNEWD~METLVGFACGHVFHLSHLLETIN--PDQPSD
NCU05902.1	THRRPKSAAKKYQAGHCAQCLSPFTTYE~TDTLVGFACGHVFHLPHLLEALN--PGKTVD
FG06075.1 	DHRVPK-------PGHCARCHEPFTEYE~METLVGYACGHVFHVSHLLEMLH--GNKKMD
AN4876.1  	TQ----------QAGRCAGCHRPFHANE1KEILVAFACGHAFHLSHVHQSEPSSPAHTPG
          	             .*:*. *  .*   :  : **.:****.**:.*: :   ::  :  .

MG03313.1 	GSEGLLNGDSF-----RATETVGGRLHVRSKVTHARLLKDKIRGGCPMCPCDRSKEM---
NCU05902.1	--ADVLLGG--------MGAEERSAHLVGAKVTHARLLRDRIVGGCVVCKAAKEEEGEGR
FG06075.1 	--IDLGNG-----------SEEGSRYSVHMKVMRARLLKDKMRGGCPVCHVKE-------
AN4876.1  	LESGVQTPRPYPPRTPNLEEPSTTSRTVGPKVTTARLLRDRIGDGCRICALAKELEAVGD
          	 . .:    . .. :.           *  **  ****:*:: .** :*   .. .  . 

MG03313.1 	---
NCU05902.1	---
FG06075.1 	---
AN4876.1  	SEA
          	:.:
```
